# Primary breast diffuse large B-cell lymphoma: a population-based study from 1975 to 2014

**DOI:** 10.18632/oncotarget.23285

**Published:** 2017-12-08

**Authors:** Yijun Jia, Chenbo Sun, Zebing Liu, Weige Wang, Xiaoyan Zhou

**Affiliations:** ^1^ Department of Pathology, Fudan University Shanghai Cancer Center, Shanghai 200032, China; ^2^ Department of Oncology, Shanghai Medical College, Fudan University, Shanghai 200032, China; ^3^ Institute of Pathology, Fudan University, Shanghai 200032, China; ^4^ Department of Pathology, Renji Hospital, School of Medicine, Shanghai Jiaotong University, Shanghai 200127, China

**Keywords:** breast lymphoma, diffuse large B-cell lymphoma, incidence, survival

## Abstract

Primary breast diffuse large B-cell lymphoma (DLBCL) is a rare non-Hodgkin’s lymphoma with limited data. In this study, a population-based study of primary breast DLBCL in the United States was performed to determine its incidence trends, prognostic factors, survival, the role of surgery as well as the comparison with nodal DLBCL. 1021 patients diagnosed with breast DLBCL were identified in the Surveillance, Epidemiology, and End Results (SEER) cancer registries from 1973–2014. The incidence of both breast and nodal DLBCL increased over time. Patients with breast DLBCL were older, mainly women, diagnosed at earlier stages and had lower prevalence in white and black races compared with nodal DLBCL. Multivariate analysis revealed older age (≥ 70 years old) and advanced stage as independent predictors of worse OS. Independent predictor of better DSS were younger age (< 70 years old), early stage and diagnosis after 2000. When analyzed according to age, stage, race, tumor laterality and year of diagnosis, the overall survival did not benefit from surgery except in patients diagnosed between 2001–2010 and the surgery rate decreased overtime. Compared with nodal DLBCL, breast DLBCL patients exhibited a better outcome. In conclusion, breast DLBCL is a rare tumor with increasing incidence and improved survival over the last four decades. The introduction of rituximab seems to improve the outcome of breast DLBCL. Further studies are needed to advance our understanding of breast DLBCL and optimize the treatment strategy.

## INTRODUCTION

Breast is a rare site of extranodal involvement of lymphoma. Breast lymphoma represents approximately 0.5% of breast malignant neoplasms and between 1.7–2.2% of extranodal lymphoma [1,2]. It was first described in 1972 by Wiseman and Liao [3] in a group of 31 patients diagnosed between 1951 and 1970. They defined it as the infiltration of breast tissue by lymphoma with or without regional lymphnode in patients without a history of prior nodal or extranodal lymphoma and systemic diseases at the time of diagnosis. The frequent clinical presentation of primary breast lymphoma is painless palpable mass which is similar to that of breast cancer. The infrequent presentation includes skin edema [4], erythema [4–6] and retraction [7]. Primary breast lymphoma is usually non-Hodgkin’s B-cell type, which accounts for about half of breast lymphomas, and the most frequent subtype is diffuse large B-cell lymphoma (DLBCL) [8–12].

As this breast malignancy is rare, there is limited information about its epidemiology and outcome and how it compares with primary nodal diffuse large B-cell lymphoma still remains unknown. Our current knowledge about breast DLBCL is based on anecdotal reports and retrospective studies with small numbers of patients. As a result, clinicians have little prospective data to guide optimal treatment.

The treatment options for primary breast lymphoma vary broadly including surgical intervention, chemotherapy and radiotherapy [13]. However, the optimal management of patients with breast lymphoma remains unclear and controversial. Chemotherapy is the mainstay of therapy based on the success of chemotherapy in the treatment of nodal and other extranodal non-Hodgkin’s lymphomas. Mastectomy for primary breast lymphoma is not well-supported because it shows neither improved survival nor reduced risk of recurrence [9, 11, 14]. The role of radiotherapy has never been explored prospectively. One of the largest studies of breast DLBCL was reported by Jennings WC et al. [14], and they searched several databases and reviewed 92 articles in which patient-specific treatment and follow-up information were included, to determine the best treatment strategies for primary breast lymphoma. In that study, 465 acceptable patients reported from 1972 through 2005 were included. The study showed that nodal status predicts survival and outcome and guided optimal use of radiation and chemotherapy.

The Surveillance, Epidemiology, and End Results (SEER) database [15] is a useful resource for analyzing rare malignancies like primary breast lymphoma in settings for which prospective data or trials are limited. The main purpose of this retrospective study based on the SEER database is to provide the best available information to improve understanding of primary breast lymphoma. In this study, we examined the incidence and survival trends of breast DLBCL and compared characteristics between patients with breast and nodal DLBCL. This study also revealed prognostic factors of breast DLBCL, the effect of surgery and the potential impact of the introduction of rituximab.

## RESULTS

### Incidence of breast DLBCL

**Figure 1:**
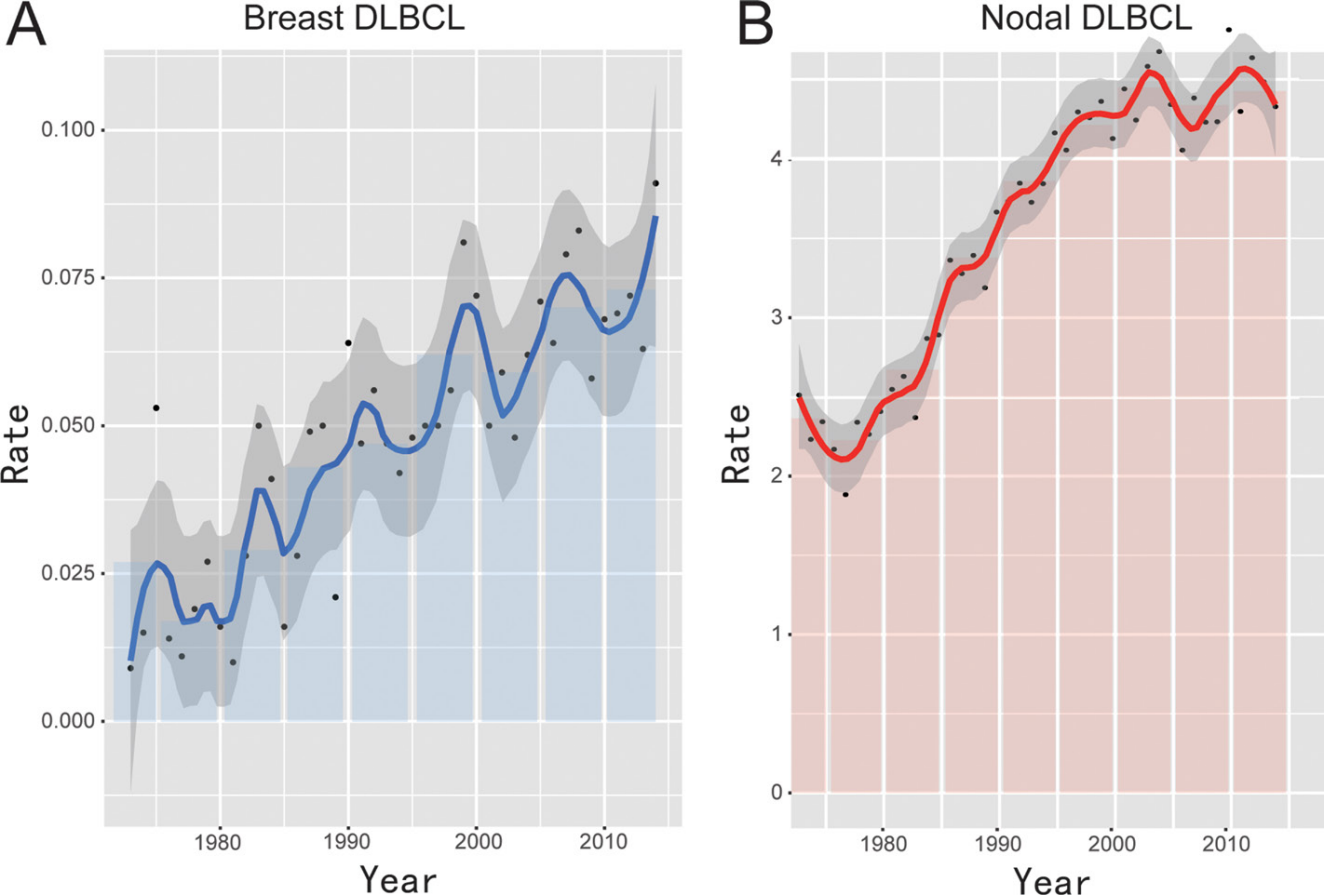
Overall incidence of breast DLBCL and nodal DLBCL from 1973 to 2013 adjusted to the 2000 standard US population

The overall incidence rate of breast DLBCL was approximately 0.052 per 100,000 (adjusted to the 2000 standard US population). The overall cancer incidence rate in breast lymphoma increased from 0.03 per 100,000 people in 1973 to 1990 to 0.073 in 2011 to 2014, with a significant trend toward increasing incidence for breast DLBCL (APC = 1.738; 95% CI = 1.9–3.6, *P* < 0.01). Figure 1A and Table 1 illustrated long-term trends in incidence rates for breast DLBCL.

**Table 1:** Incidence Trends for Breast DLBCL and Nodal DLBCL from 1973–2014

	Overall Trend			Trend 1		Trend 2		Trend 3		Trend 4		Trend 5		Trend 6	
				(1973–1990)		(1991–1995)		(1996–2000)		(2001–2005)		(2006–2010)		(2011–2014)	
	Rate	APC		Rate	APC	Rate	APC	Rate	APC	Rate	APC	Rate	APC	Rate	APC
Breast DLBCL															
Overall	0.0522	2.7^*^		0.03	5.0	0.048	-2.6	0.062	13.1	0.059	8.2	0.07	-1.9	0.073	8.5
Age															
< 70	0.026	NA		0.016	NA	0.019	0.02	0.03	7.4	0.032	0.28	0.033	-3.0	0.035	-4.6
≥ 70	0.314	NA		0.171	NA	0.327	-5.3	0.378	17.7^*^	0.321	14.3	0.44	-0.8	0.451	20.2
Race															
White	0.05	3.0^*^		0.029	3.2	0.045	-0.5	0.06	8.2	0.053	14.6^*^	0.075	-1.3	0.073	11.2
Black	0.032	NA		0.01	NA	0.018	NA	0.035	NA	0.056	-22.5	0.045	NA	0.034	NA
Other	0.082	NA		0.06	NA	0.089	NA	0.1	NA	0.089	-0.8	0.061	-12.2	0.097	6.2
sex															
Female	0.087	NA		0.052	NA	0.078	2.3	0.105	11.7	0.104	5.5	0.115	-1.6	0.122	10.1
Male	0.007	NA		0.002	NA	0.004	NA	0.006	NA	0.001	NA	0.016	NA	0.015	-9.1
Stage															
I	0.029	NA		0.013	NA	0.027	5.0	0.043	13.6	0.036	10.2	0.038	-16.1	0.038	17.8
II	0.008	NA		0.002	NA	0.007	NA	0.01	-0.3	0.012	2.9	0.012	12.2	0.015	17.1
Nodal DLBCL															
Overall	3.762	1.7^*^		2.739	3.2^*^	3.862	2.3	4.216	0.5	4.453	0.5	4.341	3.2	4.43	-0.2-
Age															
< 70	2.185		1.5^*^	1.637	3.1^*^	2.409	2.2	2.505	-0.1	2.538	-0.4	2.409	1.6	2.471	-1.6
≥ 70	19.297		2.0^*^	13.601	3.3^*^	18.176	2.3	21.073	1.3	23.33	1.4	23.385	4.9^*^	23.739	1.2
Race															
White	3.926		1.8^*^	2.882	3.4^*^	4.104	1.8	4.448	0.7	4.667	0.6	4.491	2.8	4.697	0.0
Black	2.79		2.5^*^	1.688	4.3^*^	2.515	9.8	3.015	-3.2	3.509	5.3	3.439	4.1^*^	3.469	2.7
Other	2.837		1.9^*^	1.815	-0.3	2.539	7.6	2.875	3.7	3.118	-2.9	3.628	4.6	3.081	-5.0
Sex															
Female	4.601		1.5^*^	2.359	2.3^*^	3.24	3.2	3.487	-0.6	3.634	0.3	3.521	2.9	3.544	0.1
Male	3.101		1.9^*^	3.227	4.0^*^	4.608	1.5	5.108	1.6	5.487	1.0	5.355	3.3	5.515	-0.7
Stage															
I	0.637		NA	0.305	NA	0.887	1.6	0.919	-0.9	0.921	-1.9	0.688	-0.2	0.64	-4.0
II	0.593		NA	0.273	NA	0.63	5.6	0.758	2.1	0.805	0.9	0.799	0.6	0.771	-3.6
III	0.666		NA	0.215	NA	0.663	0.8	0.781	4.8	0.891	6.2^*^	0.972	4.0	1.129	2.5
IV	1.286		NA	0.628	NA	1.447	2.8	1.534	-1.4	1.7	-0.3	1.714	4.6	1.684	2.2

Abbreviation: CI, Confidence Interval; NA, Not Applicable; APC, Annual percent change based on incidence (delay adjusted) and mortality rates age adjusted to the 2000 US standard population. ^*^The APC is significantly different from zero (*P* < 0.05).

Analysis by race showed that blacks have the lowest incidence (0.032 per 100,000). While the highest incidence rate was observed in American Indians, Alaskan natives, and Asian/Pacific Islanders (0.082 per 100,000). Incidence rates of whites were between these two groups. For whites, the incidence of breast DLBCL increased at an APC of 3.0 (95% CI = 2.1–3.8; *P* < 0.01) and there was a statistically significant increase from 2001 to 2005, with an APC of 14.6 (95% CI = 1.4–29.6, *P* < 0.01). Among age groups, the incidence rate of breast DLBCL was higher among patients of ≥ 70 years old (0.314 per 100,000) compared with that of patients with the age of < 70 years old (0.026 per 100,000). For individuals aged 70 years or older, a statistically significant increase in breast DLBCL incidence could be observed from 1996 to 2000 and the APC was 17.7 (95% CI = 3.9–33.4, *P* < 0.01). When analyzed by Ann Arbor Stage, the incidence rate for breast DLBCL diagnosed at stage I was 0.029, while the incidence rate for patients with stage II was 0.008. For females, the incidence rate for breast DLBCL was 0.087, while the incidence rate for male breast DLBCL was 0.007.

### Patient demographics and tumor characteristics

A total of 1021 patients with breast DLBCL were identified in the SEER-18 database from 1973–2014. Breast DLBCL is generally a disease of the elderly, with 70.7% patients diagnosed at > 60 years old. The median age was 70 years old. A predominance of patients were female (96.4%). Whites comprised the highest proportion of patients accounting for 81.3% of cases. The right breast was as frequently involved as the left, whereas bilateral breast involvement was not common (2.6%). Among patients for whom staging information was available, approximately 53.6% of breast DLBCL patients were categorized as stage I, and 17.5% as stage II. Surgery was used as a component of therapy in 237 patients (23.2%). The information of demographics, tumor characteristics and surgery received were summarized in Table 2.

**Table 2:** Demographic and clinical characteristics of patients with breast and nodal DLBCL

	Breast DLBCL (*n =* 1021)		Nodal DLBCL (*n =* 74440)		
Characteristic	No. of patients	Percentage (%)	No. of patients	Percentage (%)	*P*
Year of diagnosis					0.074
1973–1990	99	9.7	9207	12.4	
1991–2000	200	19.6	14411	19.4	
2001–2010	483	47.3	34374	46.2	
2011–2014	239	23.4	16448	22.1	
Age					< 0.001
<50	152	14.9	14629	19.7	
50–59	148	14.5	11591	15.6	
60–69	207	20.3	15865	21.3	
70–79	259	25.4	18350	24.7	
≥ 80	255	25	14005	18.8	
Sex					< 0.001
Male	37	3.6	40300	54.1	
Female	984	96.4	34140	45.9	
Race					< 0.001
White	830	81.3	63797	85.7	
Black	54	5.3	5334	7.2	
Other (American	130	12.7	4959	6.7	
Indian/AK Native,					
Asian/Pacific Islander)					< 0.001
Stage					
I	547	53.6	12111	16.3	
II	179	17.5	12967	17.4	
III	-	-	14687	19.7	
IV (bilateral)	146	14.3	26301	35.3	
Unknown	149	14.6	8374	11.2	
Tumor laterality					
Right	502	49.2			
Left	484	47.4			
Bilateral	27	2.6			
Surgery					< 0.001
Yes	237	23.2	11999	16.1	
No	495	48.5	41079	55.2	
Unknown	289	28.3	21362	28.7	

### Survival and prognostic factors

**Figure 2:**
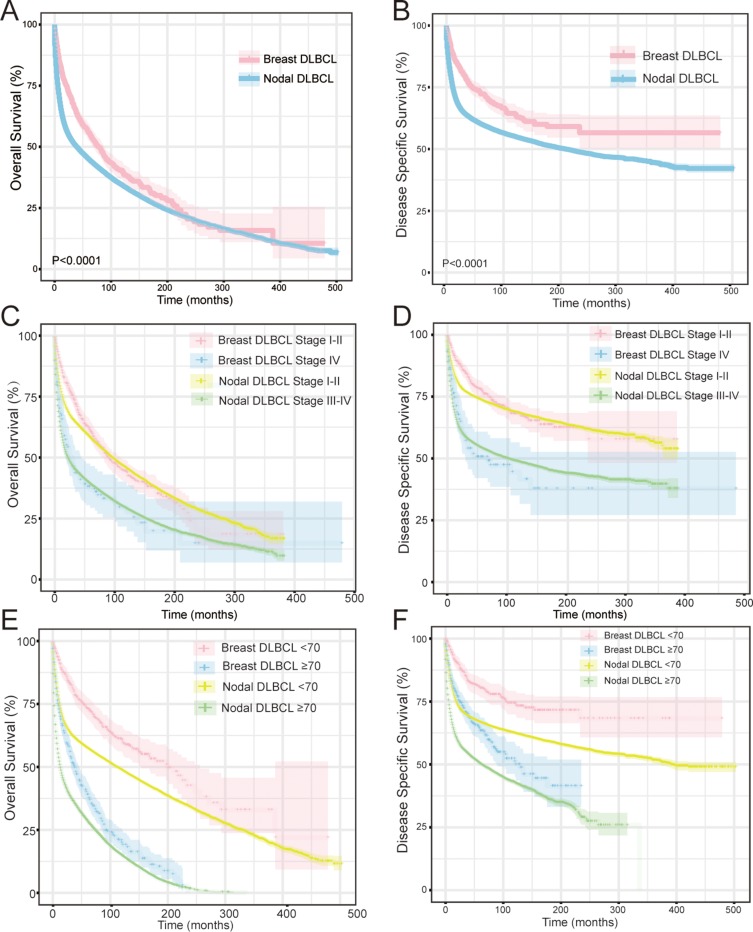
Comparison of overall survival (OS) and disease-specific survival (DSS) of breast DLBCL and nodal DLBCL (**A**), (**B**): Kaplan-Meier survival curves of OS (A) and DSS (B) in breast DLBCL and nodal DLBCL. (**C**) (**D**): Kaplan-Meier survival curves of OS (C) and DSS (D) by stage (stage I–II vs stage III–IV) in breast DLBCL and nodal DLBCL. (**E**) (**F**): Kaplan-Meier survival curves of OS (E) and DSS (F) by age (< 70 years old vs ≥ 70 years old) in breast DLBCL and nodal DLBCL.

Approximately 992 cases (97.2%) with complete survival information were eligible for inclusion in analyses of the OS and DSS of patients with breast DLBCL. OS and DSS for the entire cohort of breast DLBCL patients are presented in Figure 2A and 2B. The overall survival (OS) rates at 5, 10, 15, and 20 years for breast DLBCL was 94.6%, 89.7%, 82.5% and 75.1%, whereas DSS rates at 5, 10, 15, and 20 years were 94.7 %, 87.2%, 79.6% and 69.6%, respectively. Patients with stage I–II exhibited improved OS and DSS compared to stage IV patients (both *P* < 0.001) (Figure 2C and 2D). Younger patients (< 70 years old) showed prolonged OS and DSS compared to elderly patients (≥ 70 years old) (both *P* < 0.001) (Figure 2E and 2F).

**Figure 3:**
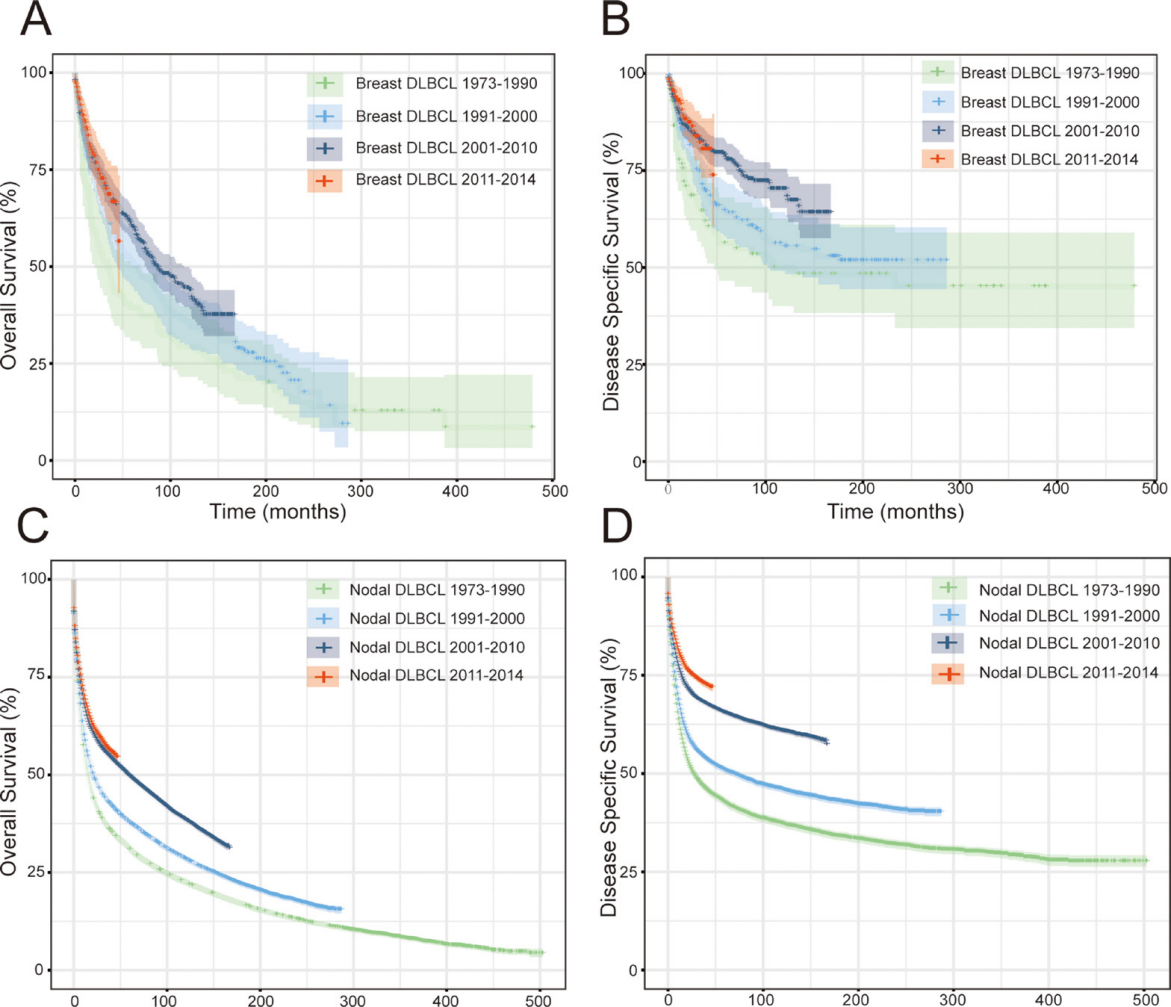
OS and DSS of breast DLBCL and nodal DLBCL according to era of diagnosis (1973–1990, 1991–2000, 2001–2010, and 2011–2014)

The OS improved significantly over time, OS for patients diagnosed in 2001 to 2010 and for patients diagnosed in 2011–2014 was both significantly improved compared with that of patients diagnosed in 1973 to 1990 (both *p* < 0.001). Patients diagnosed in 2001 to 2010 exhibited prolonged DSS compared with that of patients diagnosed in 1991–2000 (*P* = 0.004) (Figure 3A and 3B). Univariate and multivariate analyses were performed to determine independent prognostic factors predicting overall and disease-specific survival with adjustment for various clinical variables. The effects of variables on OS and DSS are listed in Table 3. Our univariate analysis revealed that older age, white races, advanced stage (IV), no surgery and diagnosis before 2000 were associated with worse overall survival. Younger age, early stage (I–II) and diagnosis after 2000 were predictors of improved DSS. In the multivariate analysis, independent predictors of better OS and DSS were younger age and early stage (I–II).

**Table 3:** Univariate and multivariate analyses of clinical characteristics associated with OS and DSS of patients with breast DLBCL

		Univariate Analysis							Multivariate Analysis						
		Overall Survival				Disease-Specific Survival			Overall Survival			Disease-Specific Survival			
Variables		Median	HR	95% CI	*P*	HR	95% CI	*P*	HR	95% CI	*P*	HR	95% CI		*P*
Age (years)															
< 70		58	1			1			1			1			
≥ 70		26	3.3	2.74–3.97	<0.001	2.33	1.82–2.98	< 0.001	3.34	2.76–4.04	< 0.001	2.34	1.82–3.00		< 0.001
Race															
White		38	1			1			1						
Others		44	0.711	0.56–0.90	0.005	0.87	0.64–1.19	0.382	0.96	0.75–1.48	0.732				
Stage															
I-II		45	1			1			1			1			
IV (bilateral)		19.5	1.91	1.52–2.40	< 0.001	2.58	1.93–3.44	< 0.001	1.93	1.54–2.44	< 0.001	2.73	2.04–3.65		<0.001
Unknown		31	1.43	1.14–1.79	0.002	1.16	0.81–1.67	0.41	1.31	1.03–1.66	0.028	1.03	0.71–1.50		0.87
Tumor Laterality															
Right		40	1.04	0.88–1.24	0.63	0.99	0.78–1.27	0.96							
Left		41	1			1									
Bilateral		30	1.239	0.72–2.13	0.44	1.61	0.82–3.17	0.17							
Surgery															
No		34	1			1			1						
Yes		64.5	0.77	0.62–0.97	0.025	0.75	0.54–1.04	0.08	0.83	0.66–1.04	0.145				
Year of diagnosis															
1973–1990	34		1			1			1			1			
1991–2000	62		0.81	0.62–1.06	0.12	0.79	0.55–1.14	0.21	0.86	0.64–1.16	0.32	0.7		0.48–1.03	0.067	
2001–2010	63		0.66	0.51–0.85	0.001	0.51	0.36–0.73	< 0.001	0.89	0.59–1.34	0.578	0.45		0.31–0.64	< 0.001	
2011–2014	14		0.62	0.43–0.89	0.009	0.48	0.30–0.79	0.003	0.88	0.54–1.44	0.619	0.44		0.27–0.72	0.001	

### Surgical intervention

**Figure 4:**
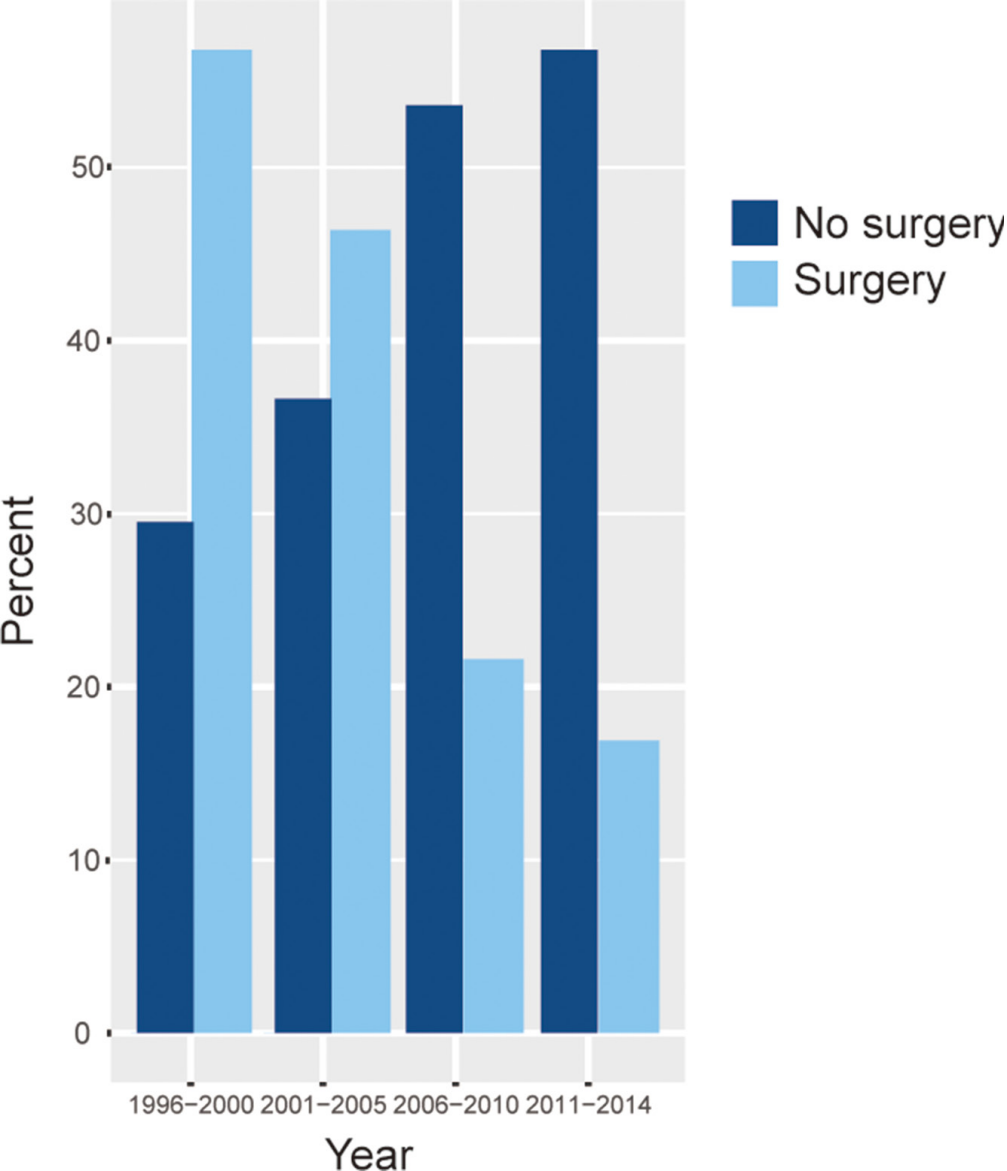
The proportions of patients with breast DLBCL that received surgery and no surgery over time.Y axis indicates percentage of patients (%), and X axis indicates the year of diagnosis (1996–2000, 2001–2005, 2006–2010 and 2011–2014)

Surgical intervention changed markedly from 1991 to 2014. From 1991 to 2000, surgery was delivered to 56.8% of patients with breast DLBCL, while 43.2% patients received no surgery. By 2001 to 2010, the proportion of patients that received surgery at diagnosis decreased to 33.0%. The percentage of surgery received by breast DLBCL patients further decreased to 16.9%. The rates of surgery and no surgery according to the era of diagnosis are shown in Figure 4.

Subgroup analyses were performed to determine the factors that correlated with the effect of surgical intervention and the *P* value of overall survival (OS) and disease specific survival (DSS) comparing surgery and no surgery were listed in Table 4. Overall, the surgery group showed improved OS in patients compared to the no-surgery group (*P* = 0.014), however, there was no statistically significant difference in DSS of patients between the two groups (*P* = 0.057). Age had no effect on surgical efficacy in breast DLBCL. Breast DLBCL patients of white who underwent surgery exhibited an improved survival rate according to Log-rank analysis, but this survival benefit was not statistically significant as the *P* value is on the critical point (*P* = 0.05). The overall survival benefit of surgery was observed in patients diagnosed between the year of 2001–2010 (*P* = 0.036). No significant survival improvement was found in the remaining cohorts with surgery.

**Table 4:** Analysis of effects of surgical treatment on OS and DSS

	Surgery		No Surgery		Overall survival	Disease-specific survival
	No. of patients	Median Months	No. of patients	Median Months	*P* value	*P* value
All	248	64.5	556	34	0.014	0.057
Age						
< 70	128	98.5	278	40	0.144	0.068
≥ 70	120	41.5	278	20	0.12	0.433
Race						
White	196	65.5	456	34	0.05	0.067
Others	52	58.5	100	34	0.137	0.589
Stage						
I-II	197	70	388	39	0.224	0.246
IV (bilateral)	25	30	97	16	0.174	0.218
Tumor Laterality						
Right	116	62	275	33	0.463	0.061
Left	128	69	257	35	0.946	0.503
Bilateral	4	51	18	15	0.958	0.641
Year of diagnosis						
1973–1990	0	NA	0	NA	NA	NA
1991–2000	50	110	38	67	0.14	0.396
2001–2010	158	77	321	58	0.036	0.08
2011–2014	40	16.5	197	13	0.8	0.38

### Comparison with nodal DLBCL

Consistent with the increase in the incidence trend of breast DLBCL, nodal DLBCL has also increased in frequency during the past four decades (Figure 1B). The increase in the incidence trend could be observed in both age groups (< 70 and ≥ 70 years old), females and males, and all the races (white, black and others) (Table 1). A total of 74440 patients with nodal DLBCL were identified. Patients with breast DLBCL were older, mainly women, diagnosed at earlier stages and had lower prevalence in white and black races compared with nodal DLBCL (Table 2).

Patients with breast DLBCL had better DSS than that of nodal DLBCL (*P* < 0.0001). The median OS of breast DLBCL was also better than that of nodal DLBCL for the first 18 years, although the curves with 95%CI overlapped afterwards (*P* < 0.0001) (Figure 2A and 2B). When patients were categorized according to stage, breast DLBCL seemed to have better OS initially for I-II stage, however this advantage disappeared beyond 6 years (*P* = 0.118). Regarding DSS, breast DLBCL with stage I–II had a better outcome than nodal DLBCL with stage I–II (*P* = 0.041). There was no significant difference between nodal and breast DLBCL for advanced stage (stage III–IV in nodal DLBCL vs. Stage IV in breast DLBCL) in OS and DSS (*P* = 0.628 and *P* = 0.969) (Figure 2C and 2D). When we examined survival according to age, no matter 70 years and older or 70 years younger, OS and DSS of breast DLBCL were always better than nodal DLBCL (all *P* < 0.001) (Figure 2E and 2F)

When analyzing survival of nodal DLBCL by the era of diagnosis, we found that OS and DSS improved significantly over time which is more obvious than breast DLBCL. OS and DSS for patients diagnosed in 1991 to 2000 was significantly improved compared with that of patients diagnosed in 1973 to 1990. Patients diagnosed in 2001 to 2010 exhibited prolonged OS and DSS compared with that of patients diagnosed in 1991 to 2000. Significant OS and DSS improvements was also found in patients diagnosed in 2011 to 2014 and patients diagnosed in 1991 to 2000 (all *P* < 0.001). (Figure 3C and 3D).

## DISCUSSION

To our knowledge, this is the first epidemiological study and the largest population-based study describing the incidence, characteristics and management of breast DLBCL in the United States based on the SEER database. There has been a similar study from United States [16], however their eligibility included all types of breast lymphoma and investigated mainly on their incidences and survivals. We retrieved the newest data released on 28/4/2017 and included the patients diagnosed from the year of 1973 to 2014. We focused on breast DLBCL, which is the most frequent subtype of breast lymphoma and expanded on their work to study breast DLBCL in several ways, such as baseline characteristics, prognostic factors, comparison with nodal DLBCL as well as the role that surgery plays in the treatment of breast DLBCL.

Several important conclusions can be derived based on our results. We observed that the incidence rate of breast DLBCL, especially in whites (APC = 3.0; 95% CI = 2.1–3.8), is increasing overtime, which may be partly due to the increased awareness of the disease as a unique entity by pathologists and clinicians over time. Likewise, we also found a similar increase in the incidence trend of nodal DLBCL.

Consistent with previous studies [6,17], our study indicates a nearly exclusive incidence in females. It suggests a potential role for sex hormones in the pathogenesis of primary breast lymphoma. Epidemiological data suggested that estrogen was a risk factor for lymphoma [18–20], and a recent large study found that women treated with estrogen hormone replacement therapy had 29% increased risk of developing non-Hodgkin’s lymphoma (not specifically primary breast lymphoma) compared with women that never exposed to [21].

Bilateral involvement of the breast appears to be a feature of aggressive disease with poor prognosis [9, 22]. Previously published studies [9, 23] reported a right-sided predisposition for breast DLBCL. As the cohort became larger, the right-sided predominance became less apparent. In our study, we showed balanced laterality of breast involvement at the time of diagnosis (right: left = 1.04:1). We also observed that 2.6% patients were diagnosed with bilateral breast DLBCL, which is consistent with the other two reports that showed involvement of bilateral breast in 1% and 5% patients [9, 23].

Age at diagnosis was an important predictor of survival in the multivariate analysis and younger patients ( < 70 year old ) exhibited improved survival outcomes. This findings could be observed in both breast and nodal DLBCL. Early tumor stage was also associated with improved survival in breast DLBCL as well as nodal DLBCL. Univariate analysis also showed significant OS benefits in non-white races and surgery group, however, the benefits were lost in multivariate analysis. It is unclear whether this observation is due to distinct tumor biology or to socioeconomic factors, further investigation is needed.

Increasing trends of overall survival and disease-specific survival were observed over time, which may be due to improvements in the comprehensive approach of treatment. Particularly, the rapidly developing targeted therapy for CD20-positive B lymphoma has further improved survival in patients with lymphoma [24–27, 35]. Data from our study also provided a comparison of the treatment of breast DLBCL before and after the rituximab era in a population. We found a significant improvement in DSS for breast DLBCL and in both OS and DSS for nodal DLBCL after the introduction of rituximab.

As the rarity of breast DLBCL, data are limited. With the rise of breast DLBCL, management strategies have been revisited. However, the treatment recommendations are difficult to make and the role of surgery in this comprehensive breast DLBCL therapy strategy is still subject to debate. Excisional biopsy should be performed to facilitate correct diagnosis. Although fine needle aspiration may differentiate carcinoma from lymphoma, it lacks details that are necessary to accurately classify subtypes of NHL and thus is insufficient as a diagnostic procedure [30]. Previous data showed that surgical resection results in inferior local control [19], and some studies demonstrated that treatment including mastectomy is associated with higher all-cause and disease-specific mortality [9, 14, 31]. Our results showed that the proportions of patients received surgery decreased over time. This may be largely due to complications of surgery which leads to higher mortality [9, 14, 31], and the use of anthracyclines-based chemotherapy and rituximab which have been shown to have a beneficial effect on PFS and OS [1, 9, 24–29, 32, 35]. In the present results, an OS benefit from surgery was noted in overall patients, but the benefit of DSS was lost. Patients diagnosed between the year of 2001 to 2010 with surgery showed improved overall survival compared with no surgery group. A possible and plausible explanation for this finding is that patients who had surgery might have received combined therapy that involves chemotherapy and radiotherapy which is a current treatment approach for DLBCL. When analyzed according to age, stage, race, tumor laterality, year of diagnosis, the overall survival benefit of surgery could only be found in patients diagnosed between the year of 2001–2010 (*P* = 0.036). No OS or DSS benefit of surgery was observed in other groups. Thus surgical intervention beyond excisional biopsy was not recommended.

We found that as a group, breast DLBCL had a better OS and DSS. The major reason for this finding may be that breast DLBCL predominantly presents as early stage disease compare with nodal DLBCL (71.1% vs 33.4%). As most lesions of breast DLBCL are in superficial anatomic location, they can be detected by screening mammography in an early phase. This could also be explained by the our results that no OS advantage in patients with I-II stage breast DLBCL was found compared to their nodal counterparts when patients were categorized according to stage. The distinction of clinical characteristics between breast DLBCL and nodal DLBCL could be observed in our study. Classification and management of breast DLBCL may be revisited as an entity, similar to CNS and cutaneous DLBCL.

There are several potential limitations to consider with the present study. Firstly, the treatment information that is available is limited to surgery, since SEER-18 released on 28/4/2017 also lacks information on the use of radiotherapy and chemotherapy. It was not possible to know what proportions of patients actually received anthracycline-based chemotherapy, rituximab and CNS prophylaxis now routinely used in clinical practice of DLBCL. As a result, several potential prognostic factors were not considered in this analysis. However, based on our clinical practice and previous published data [23], it is plausible to speculate that most patients did undergo some form of chemotherapy appropriate for DLBCL and many, if not most, patients did receive immunochemotherapy with rituximab. Secondly, although SEER has strict categorization guidelines, the stage information of some patients may be wrong. However, most of the patients were staged correctly, since the number of patients with stage III and nonbilateral stage IV was limited. Those cases were excluded from our analyses. Thirdly, detailed information on the type of surgery performed, margin status, peri-operative complications is not available in SEER database. Furthermore, the inherent limitations of using a population-based database including variations in data reporting and coding system, patient migration, and selection bias, might potentially exist in this study. Given above, the results of this analysis should be interpreted with caution. Despite these limitations, SEER database remains a valuable source in studying such kind of rare cancers. This study can still offer important insights for breast DLBCL, and provide useful information on baseline characteristics and outcomes.

Prospective studies should be performed to confirm these findings and future work in a multinational collaborative way is needed to advance our understanding of the etiology and biology for this rare malignancy. Results can then be used to develop evidence-based treatment recommendations and guidelines for clinical practitioners.

## MATERIALS AND METHODS

National Cancer Institute’s Surveillance, Epidemiology, and End Results (SEER) database collects and publishes cancer incidence and survival data from population-based cancer registries covering approximately 28% of the US population [15]. Nine registries including Atlanta, Connecticut, Detroit, Hawaii, Iowa, New Mexico, San Francisco-Oakland, Seattle-Puget Sound, and Utah were included in SEER-9 before 1992. Since 1992, nine more registries were added sequentially overtime, including Los Angeles, San Jose-Monterey, Rural Georgia, the Alaska Native Tumor Registry, Greater California, Kentucky, Louisiana, New Jersey and Greater Georgia to become the current SEER-18. We queried SEER-18 Registries Research Data, Nov 2016 (released on 28/4/2017) using SEER Stat 8.3.4 software (National Cancer Institute, Bethesda, MD; available at http://seer.cancer.gov/seerstat/) (accessed May 2017) and identified patients diagnosed with DLBCL with breast or lymph node listed as the primary site. International Classification of Disease for Oncology, third edition histology codes (ICD-O-3) for DLBCL (9680, 9675, 9684) were used to identify all patients with DLBCL. Codes C50.0–50.9 and codes C77.0–C77.9 were used to identify patients with breast or lymph nodes as the primary site, respectively. Patients were excluded if diagnosis was made on the death certificate or at autopsy. Data on patient demographics, stage at diagnosis, laterality of breast involvement, treatment of surgery or radiotherapy received, survival data, and cause of death were extracted from this database. There is no data available in SEER regarding the use of chemotherapy or indications for surgery. Patient characteristics were summarized using descriptive statistics and continuous variables were converted into categorical variables including age and year of diagnosis. Tumors were staged according to the Ann Arbor Stage classification for lymphoma [33]. Differences in demographics and tumor characteristics between nodal and breast DLBCL were examined using the Pearson Chi-square test and the Wilcoxon rank sum test.

All population rates are age-adjusted to the standard population of the United States in 2000. Age-adjusted incidence rate calculations were obtained from SEER-9 database (released in April 2017) and were analyzed using the SEER Stat 8.3.4. The data was expressed per 100,000 persons.

The advent of therapeutic monoclonal antibodies such as rituximab, which is approved by FDA in 1997, has increased the effectiveness of treatment for Non-Hodgkin’s lymphoma [34]. The standard of care for DLBCL which is the combination of rituximab, cyclophosphamide, doxorubicin, vincristine and prednisone was established by Coiffier B et al. from Hospices Civils de Lyon and the Université Claude Bernard in France and their study results were first presented as an abstract in December 2000 at the American Society of Hematology annual meeting [35]. As a result, we used the year of 2000 as cutoff point when we studied the potential impact of the introduction of rituximab on survival.

We partitioned surgical interventions into surgery and no surgery, to investigate the relationship between surgery and survival outcomes.

Follow-up data were up to date until 2014. Survival data was analyzed according to overall survival (OS), disease-specific survival (DSS) using Kaplan-Meier method and survival curves were constructed based on primary sites, age at diagnosis, Ann Arbor Stage and year of diagnosis. Univariate analysis using Cox-proportional hazards model was performed to evaluate the association between variables and OS/DSS. Statistically significant covariates identified in univariate analyses were included in multivariate models.

Statistical analysis was performed using SPSS statistical software version 20.0 (SPSS Inc., IBM Corporation, Chicago, IL, USA) and R version 3.3.3 Software (R Foundation for Statistical Computing, Vienna, Austria; available from http://www.r-project.org/index.html ). *P*-values < 0.05 were considered statistically significant. All confidence intervals were set as 95% CI.
